# Volatile anesthetics for lung- and diaphragm-protective sedation

**DOI:** 10.1186/s13054-024-05049-0

**Published:** 2024-09-01

**Authors:** Lukas M. Müller-Wirtz, Brian O’Gara, Marcelo Gama de Abreu, Marcus J. Schultz, Jeremy R. Beitler, Angela Jerath, Andreas Meiser

**Affiliations:** 1 Department of Anesthesiology, Outcomes Research Consortium, Cleveland Clinic, Cleveland, OH USA; 2 Department of Anesthesiology, Intensive Care and Pain Therapy, Faculty of Medicine, Saarland University Medical Center and Saarland University, Homburg, Saarland Germany; 3 Department of Anesthesiology, Friedrich-Alexander-Universität Erlangen-Nürnberg, University Hospital Erlangen, Erlangen, Germany; 4 Department of Anesthesia, Critical Care, and Pain Medicine, Beth Israel Deaconess Medical Center, Boston, MA USA; 5 Division of Intensive Care and Resuscitation, Department of Anesthesiology, Cleveland Clinic, Cleveland, OH USA; 6 Division of Cardiothoracic Anesthesiology, Department of Anesthesiology, Cleveland Clinic, Cleveland, OH USA; 7 Department of Intensive Care, Amsterdam University Medical Center, Amsterdam, The Netherlands; 8 Department of Anesthesiology, Intensive Care Medicine and Pain Medicine, Division of Cardiac Thoracic Vascular Anesthesia and Intensive Care Medicine, Medical University of Vienna, Vienna, Austria; 9 Columbia Respiratory Critical Care Trials Group, New York-Presbyterian Hospital and Columbia University, New York, NY USA; 10 Department of Anesthesiology and Pain Medicine, Temerty Faculty of Medicine, University of Toronto, Toronto, ON Canada; 11 Department of Anesthesia, Sunnybrook Health Sciences Centre, Toronto, ON Canada

**Keywords:** Intensive care, Inhaled sedation, Volatile anesthetics, Ventilation, Lung, Diaphragm, Lung- and diaphragm-protective sedation, Ventilator weaning

## Abstract

This review explores the complex interactions between sedation and invasive ventilation and examines the potential of volatile anesthetics for lung- and diaphragm-protective sedation. In the early stages of invasive ventilation, many critically ill patients experience insufficient respiratory drive and effort, leading to compromised diaphragm function. Compared with common intravenous agents, inhaled sedation with volatile anesthetics better preserves respiratory drive, potentially helping to maintain diaphragm function during prolonged periods of invasive ventilation. In turn, higher concentrations of volatile anesthetics reduce the size of spontaneously generated tidal volumes, potentially reducing lung stress and strain and with that the risk of self-inflicted lung injury. Taken together, inhaled sedation may allow titration of respiratory drive to maintain inspiratory efforts within lung- and diaphragm-protective ranges. Particularly in patients who are expected to require prolonged invasive ventilation, in whom the restoration of adequate but safe inspiratory effort is crucial for successful weaning, inhaled sedation represents an attractive option for lung- and diaphragm-protective sedation. A technical limitation is ventilatory dead space introduced by volatile anesthetic reflectors, although this impact is minimal and comparable to ventilation with heat and moisture exchangers. Further studies are imperative for a comprehensive understanding of the specific effects of inhaled sedation on respiratory drive and effort and, ultimately, how this translates into patient-centered outcomes in critically ill patients.

**Graphical abstract:**

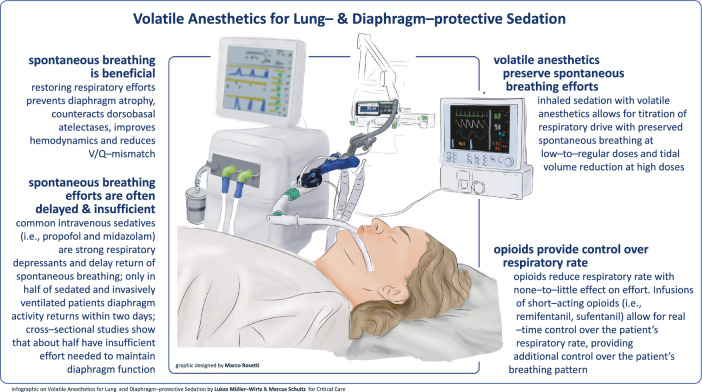

## Introduction

An early return of spontaneous breathing in invasively ventilated critically ill patients may prevent diaphragm disuse atrophy and expedite liberation from the ventilator [1–3]. However, overly vigorous respiratory efforts may induce potentially injurious high lung stress and strain, referred to as patient self-inflicted lung injury (P-SILI) [4]. Sedation and analgesia have substantial effects on respiratory drive and effort, yet their effects on outcomes of ventilated critically ill patients remain relatively unexplored [5, 6].

Until recently, sedation in invasively ventilated critically ill patients was restricted to the administration of intravenous sedatives, such as benzodiazepines, dexmedetomidine, ketamine, or propofol, each of which has relevant side effects and contraindications. Inhaled sedation with volatile anesthetics has gained popularity as an alternative to intravenous sedatives in intensive care unit (ICU) patients. Inhaled sedation may be particularly helpful for achieving lung- and diaphragm-protective *ventilation* or more specifically lung- and diaphragm-protective *sedation* [7, 8]. This concept aims to integrate the contributions of mechanical ventilation, spontaneous breathing effort, and patient–ventilator interactions to protect against iatrogenic or self-inflicted injury to the respiratory system — both the lungs and respiratory muscles. Sedation strategies play a pivotal role in lung and diaphragm protection because of their effect on respiratory drive and effort.

In this review, we explore the potential benefits of inhaled sedation for achieving lung- and diaphragm-protective sedation. We begin by introducing risks and benefits of spontaneous breathing and the relevance of sedation for lung and diaphragm protection in invasively ventilated patients. Next, we provide a synthesis of current evidence on how inhaled sedation with volatile anesthetics may help to protect the lungs and diaphragm through its effects on respiratory drive and effort. Finally, we address the technical limitations of inhaled sedation in the ICU setting.

## Methods

This is an expert opinion-based narrative review. References were thus included based on the authors’ subjective judgement on relevance to the field of research. Before synthesizing current evidence, the authors’ literature fundus was updated by searching PubMed with combinations of the following terms: volatile, inhaled, sedation, spontaneous breathing, spontaneous ventilation, respiratory drive, and lung- and diaphragm-protective ventilation. We additionally screened forward and backward citations of high-impact publications.

### Risks and benefits of spontaneous breathing

Vigorous respiratory efforts can worsen or may even induce lung injury, often referred to as ‘patient self-inflicted lung injury’ (P-SILI) [4]. P-SILI may result from high tidal volumes and breath stacking dyssynchrony [9], although the latter has recently been challenged in a porcine model [10]. Forceful inspiratory effort may alter ventilation distribution and contribute to regional overdistension from pendelluft [11–13]. Forceful exhalation may lead to alveolar derecruitment below functional residual capacity, potentially predisposing patients to atelectrauma [14]. Consistently, high driving pressure, as a surrogate for increased lung strain, is associated with adverse outcomes in assisted spontaneously breathing critically ill patients [15, 16]. Although clinical evidence for the existence of P-SILI remains indirect, it seems prudent and highly biologically plausible to reduce excessive respiratory efforts, especially in patients with injured lung tissue.

On the other hand, complete cessation of spontaneous breathing in invasively ventilated patients is detrimental to the diaphragm. Only 18 to 69 h of diaphragm inactivity under controlled mechanical ventilation results in marked diaphragm atrophy [17]. More specifically, diaphragmatic inactivity induces contractile weakness, ultrastructural fiber injury, and proteolysis in diaphragm tissue [18, 19]. In turn, excessive inspiratory effort can cause load-induced diaphragmatic injury, as shown in ultrasound studies on diaphragm thickness during invasive ventilation [3]. Both disuse atrophy and load-induced injury of the diaphragm are associated with prolonged ventilation time and ICU length of stay [20, 21]. Consequently, inspiratory efforts equivalent to those in healthy subjects at rest promise the highest probability for ventilator liberation [20]. In addition, a greater proportion of time spent at spontaneous ventilation is associated with faster liberation from the ventilator, highlighting the importance of preserving spontaneous breathing efforts during invasive ventilation [1, 2].

**Fig. 1:**
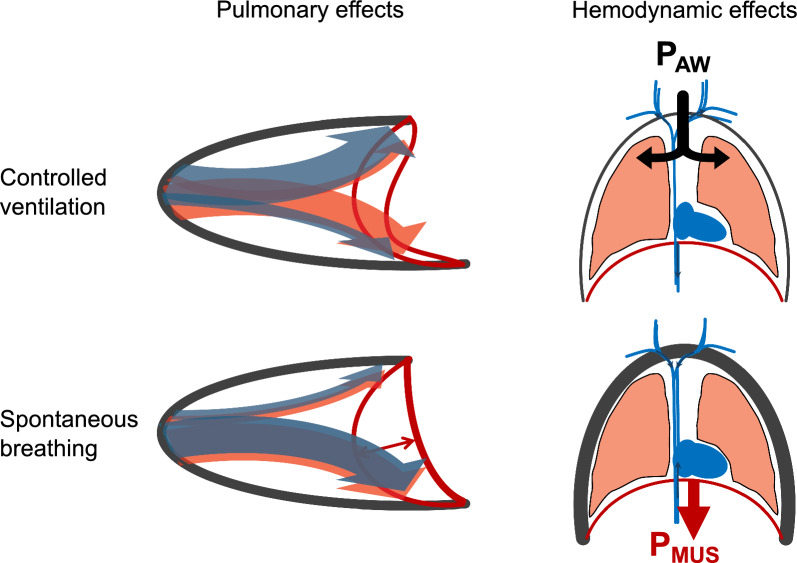
Pulmonary and hemodynamic effects of controlled ventilation and spontaneous breathing. During spontaneous breathing (lower left), contraction of the diaphragm will direct ventilation (blue arrows) to the dorsal lung regions where perfusion (orange arrows) is higher than in the ventral regions. This reduces the ventilation-perfusion mismatch which is more common in controlled ventilation (upper left). Controlled ventilation also increases intrathoracic pressures which will decrease venous return and cardiac output (upper right). Spontaneous breathing attenuates this deleterious hemodynamic effect by decreasing intrathoracic pressures during inspiration (lower right). P_AW_, airway pressure generated by the ventilator; P_MUS_, pressure generated by the respiratory muscles

In addition to the importance of spontaneous breathing for maintaining diaphragm function, it reduces ventilation heterogeneity, thereby improving ventilation-perfusion mismatch and reducing overdistension in nondependent lung regions [22, 23]. Lower intrathoracic pressures further improve hemodynamics, as evident from reduced utilization of vasopressors and better renal and hepatic perfusion during spontaneous breathing [23–25] (Fig. 1).

### Monitoring of respiratory effort

Although extremes of respiratory effort, both high and low, may contribute to lung and diaphragm injury, inspiratory effort is rarely monitored in routine clinical care of ventilator-dependent patients. Insufficient effort is twice as common as excessive effort, with roughly half of invasively ventilated patients having insufficient effort needed to maintain diaphragm function, compared to one-fourth with excessive effort [3, 20, 26, 27]. Excessive effort can be a sign of inadequately low ventilatory assistance, sedation, or analgesia, while insufficient effort often indicates ventilatory overassistance or undue sedative/analgesic effects [8]. Sedation scales poorly correlate with inspiratory effort, as even unresponsive patients may exhibit high effort, while easily arousable patients may show low or no effort at all [26]. Thus, monitoring respiratory drive and effort is necessary to ensure that spontaneous breathing is safe.

Various measures of respiratory drive and effort have been proposed alongside traditional arousal scales as targets for lung- and diaphragm-protective ventilation and sedation [7, 8]. Occlusion pressures generated during the initial 100 ms of inspiration (P_0.1_) or during an end-expiratory hold (P_occ_) are the most broadly applicable measures as required functions are integrated in most ventilators. P_0.1_ more closely relates to drive and P_occ_ to effort, while both have reasonable to excellent diagnostic accuracy for extremes of lung stress and diaphragmatic inspiratory effort [28–30]. Esophageal manometry remains the gold standard for evaluating respiratory effort but is not widely available [31]. Surface electromyography of respiratory muscles correlates reasonably well with esophageal pressure-derived measures but remains experimental [32].

In summary, the importance of restoring and preserving spontaneous breathing in invasively ventilated critically ill patients is increasingly recognized. Although monitoring of inspiratory efforts may help to increase the safety of spontaneous breathing, it is not routinely implemented.

### The concept of lung- and diaphragm-protective sedation

The ideal sedative agent would ensure patient comfort while normalizing respiratory drive and effort for maintaining diaphragm function. At the same time, it should be capable of avoiding high lung stress and strain or load-induced diaphragmatic injury. As shown in a recent physiological systematic review and in a vast number of preclinical and clinical investigations, the effects of sedatives on respiratory patterns vary substantially [5, 6]. Thus, there may not be one standard sedative that is suitable on its own to ensure adequate respiratory drive and effort for the full bandwidth of respiratory patterns in critically ill patients.

**Fig. 2:**
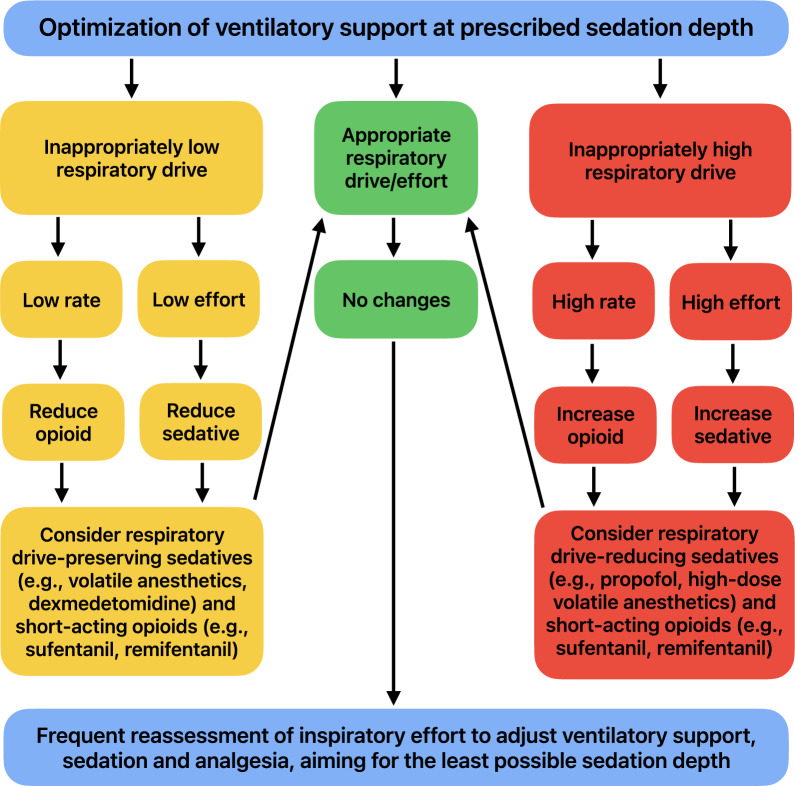
Concept of lung- and diaphragm-protective sedation

The core concept of lung- and diaphragm-protective sedation is that both respiratory drive and patient comfort are considered when choosing the sedative agent and its dose. Particularly in patients with inappropriately low or high inspiratory effort after the optimization of ventilatory assistance at the prescribed sedation depth, the sedation strategy, including agent and dose, should be reconsidered. In addition, multimodal analgesia should be leveraged to minimize the need for high doses of sedatives in line with current guidelines [33]. Opioids mostly reduce the respiratory rate with limited effects on inspiratory effort [5, 6, 34]. Utilization of short-acting opioids (e.g., sufentanil, remifentanil) in invasively ventilated patients thus improves control over respiratory rate in spontaneously breathing patients while allowing rapid correction of overdoses to restore spontaneous breathing **(**Fig. 2**)**.

Although supported by indirect evidence, there are currently no clinical trial data supporting any particular sedative strategies targeting respiratory drive and effort to improve patient-centered clinical outcomes. Lung- and diaphragm-protective sedation emphasizes individualized sedation strategies targeting safe ranges for both sedation depth and respiratory effort, thus rejecting a “one-sedative-fits-all” approach and calling for further research in this area.

### Inhaled sedation preserves respiratory drive

Several factors can modulate respiratory drive, broadly classified as biochemical inputs (pH, carbon dioxide, oxygen), mechanical inputs (lung and chest wall mechanoreceptors), suprapontine inputs (pain, discomfort, anxiety, wakefulness), and possibly inflammatory inputs [35]. Respiratory drive can be roughly divided based on the most important stimuli: wakefulness, hypoxic, and hypercapnic drive. While sedation suppresses all of these factors to some extent, wakefulness and hypoxic drive are largely eliminated by sedation and supplemental oxygen, leaving arterial pH and carbon dioxide as the major physiological determinants of respiratory drive in sedated spontaneously breathing patients [36]. Although the effects of anesthetics on ventilation may be extrapolated from perioperative clinical data, there is a scarcity of clinical investigations in critically ill patients [5].

**Fig. 3:**
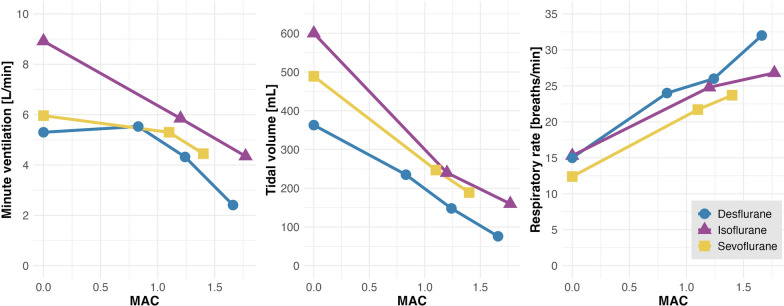
Effects of volatile anesthetics on spontaneous ventilation. All modern volatile anesthetics exert similar dose-dependent effects on respiratory parameters, with decreases in tidal volume and concurrent increases in respiratory rate. Mean values are presented. MAC, minimum alveolar concentration. The data were extracted from previous studies performed in healthy volunteers and patients scheduled for surgery [37–39]

Volatile anesthetics reduce tidal volumes and simultaneously increase respiratory rate in a dose-dependent fashion [37–39], thus bearing the potential to reduce lung stress and strain in spontaneously breathing patients **(**Fig. 3**)**. Notably, volatile anesthetics significantly suppress minute ventilation only at doses around and above 1 MAC [6], which is higher than the approximate dose of 0.5 MAC needed for intensive care sedation [40]. However, a pharmacodynamic study in 9 healthy volunteers demonstrated that sevoflurane and alfentanil synergistically decrease minute ventilation [41]. This suggests that the typical doses of volatile anesthetics used for sedation in intensive care settings are suitable to reduce excessive respiratory drive when opioids are co-administered.

On the other hand, volatile anesthetics better preserve respiratory drive than common intravenous alternatives. Proper functioning of chemosensitive brainstem neurons, particularly those in the retrotrapezoid nucleus expressing Phox2b, plays a vital role in maintaining spontaneous breathing during sedation [42]. Interestingly, preclinical experiments showed that the volatile anesthetics isoflurane and sevoflurane enhance, whereas propofol suppresses the excitability of these neurons [43, 44]. Consistently, both volatile anesthetics induce less respiratory depression than equipotent doses of propofol in animals and healthy human subjects [45–47]. Further studies with healthy volunteers showed that subanesthetic concentrations of isoflurane and sevoflurane (0.1 minimum alveolar concentration (MAC)) significantly inhibit hypoxic drive but leave hypercapnic drive largely unaffected [48–51].

**Fig. 4:**
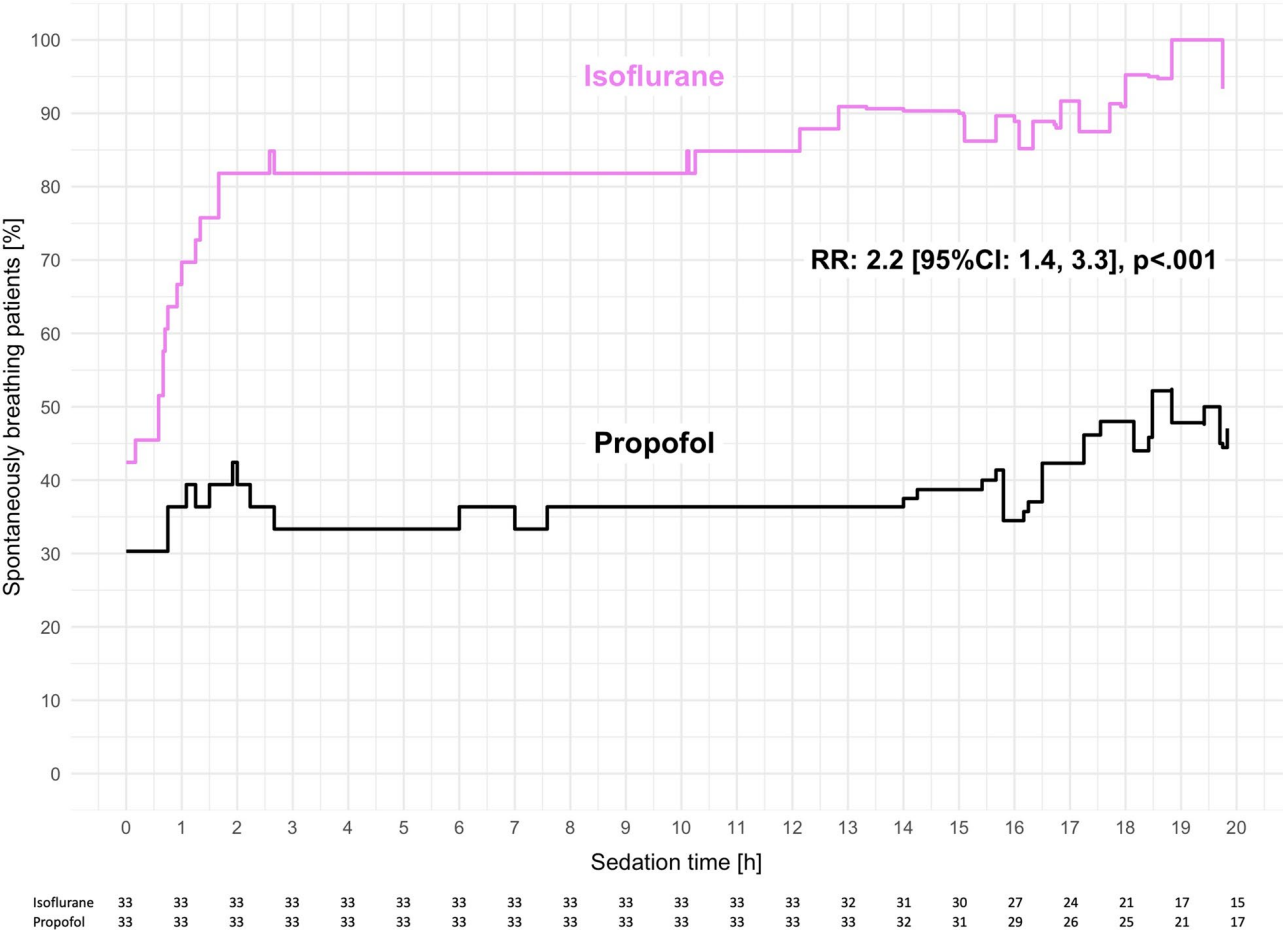
Percentage of spontaneously breathing patients over the first 20 h after randomization to isoflurane or propofol in a substudy of the Sedaconda trial. Numbers at the bottom of the figure represent the total patients included at the respective time points on the x-axis. The given risk ratio (RR) describes the effect of isoflurane versus propofol sedation on spontaneous breathing and is adjusted for sufentanil dose and arterial carbon dioxide partial pressure. 95%CI, 95% confidence interval. *Reprinted under the terms of the Creative Commons Attribution License from* [52]

To date, the largest randomized clinical trial comparing isoflurane to propofol sedation in critically ill patients –– the Sedaconda trial –– found that 50% of patients sedated with isoflurane were spontaneously breathing on day one versus 37% with propofol sedation (isoflurane n = 150, propofol n = 151; odds ratio: 1.7 [95% CI: 1.1, 2.6], *p* = 0·013) [40]. The corresponding subgroup analysis including 66 patients from a center with standards aiming at facilitation of early spontaneous breathing reported twice the probability of assisted spontaneous breathing within the first 20 h after randomization to isoflurane versus propofol (risk ratio: 2.4 [95% CI: 1.5, 3.7], *p* < 0.001) [52] **(**Fig. 4**)**. One may argue that higher arterial carbon dioxide pressures resulting from increased dead space ventilation with volatile anesthetic administration devices or opioid sparing effects have contributed [40, 53–57]. However, a mediation analysis supported that better preservation of spontaneous breathing was a direct drug effect of isoflurane independent of indirect effects mediated through increases in arterial carbon dioxide or a reduction in opioid utilization (mediator-adjusted risk ratio: 2.2 [95% CI: 1.4, 3.3], *p* < 0.001) [52].

In addition, retrospective studies and case series showed that spontaneous breathing activity is well-preserved under inhaled sedation. In a retrospective cohort study involving 38 patients who underwent continuous lateral rotational therapy, isoflurane sedation more often preserved spontaneous breathing efforts than did propofol or midazolam [58]. In a case series of 62 patients with moderate to severe acute respiratory distress syndrome, spontaneous breathing efforts were preserved 91% of the time in prone position, despite deep sedation with sevoflurane [59]. Similarly, a high proportion of time spent at assisted spontaneous breathing (96%) was reported in 15 prone-positioned COVID-19 patients sedated with a combination of sevoflurane and ketamine [60]. A comprehensive list of clinical studies reporting data on the effects of inhaled sedation with volatile anesthetics on spontaneous breathing in critically ill patients is presented in Table 1.

**Table 1:** Clinical studies reporting effects of inhaled sedation on spontaneous breathing in critically ill patients

References	Design	Sedatives	Duration	N	Percentage of spontaneously breathing patients	Specifics
Soukup [57]	RCT	Sevoflurane vs. propofol/midazolam	> 48 h Max: 183 h	79	n/a	Reduced time to spontaneous breathing after discontinuation: 26 vs. 375 min, *p* < 0.001
Bansbach [60]	Retrospective case series	Sevoflurane + esketamine	Mean: 174 h	15	100%	COVID-19 ARDS, spontaneous breathing during 96% of prone position time
Müller-Wirtz [88]	Retrospective cohort study	Isoflurane vs. propofol	> 48 h Max: 179 h	64	n/a	Isoflurane tripled the probability of increased respiratory drive after discontinuing prolonged sedation: 31 vs. 12%; RR: 2.9 [95%CI 1.3, 6.5], *p* = 0.010
Müller-Wirtz [52]	Subgroup of RCT (Meiser 2021)	Isoflurane vs. propofol	First 20 h after randomization	66	94 vs. 58%	Isoflurane doubled the probability of spontaneous breathing: RR: 2.2 [95%CI 1.4, 3.3], *p* < 0.001
Meiser [40]	RCT	Isoflurane vs. propofol	Max: 54 h	301	50 vs. 37%	Large RCT reporting differences in spontaneous breathing rates across multiple centers
Heider [59]	Retrospective case series	Sevoflurane	> 24 h Mean: 70 h	62	100%	Severe ARDS, spontaneous breathing during 91% of prone position time
Meiser [58]	Retrospective cohort study	Isoflurane vs. propofol/midazolam	> 24 h	38	90 vs. 16%	Severe ARDS, continuous lateral rotation
Meiser [87]	Retrospective case series	Isoflurane	24 h	6	100%	Severe ARDS, ECMO therapy

*RCT* Randomized Controlled Trial, *ECMO* Extra Corporal Membrane Oxygenation, *ARDS* Acute Respiratory Distress Syndrome, *RR* Risk Ratio

In essence, the mechanistic understanding and clinical evidence suggest that inhaled sedatives better preserve respiratory drive than the common intravenous alternatives propofol and midazolam, even in a setting of moderate to deep sedation requirements. At the same time, higher doses of volatile anesthetics are capable of reducing tidal volumes with compensatory increases in respiratory rate. This suggests that inhaled sedation might facilitate the titration of respiratory drive to maintain sufficient inspiratory effort at lower doses while reducing lung stress and strain at higher doses when clinically indicated. However, it remains to be determined whether volatile anesthetics are suitable for adequately controlling respiratory drive in critically ill patients with extremes of inspiratory effort, particularly in patients with acute respiratory failure.

### Inhaled sedation may facilitate ventilator liberation

Diaphragm function is fundamental for liberation from the ventilator. A single day of diaphragmatic inactivity under mechanical ventilation induces significant diaphragm atrophy with rapid progression throughout longer ventilation periods [17–19]. After the initiation of invasive ventilation, sedation impedes the return of spontaneous breathing, with diaphragm activity returning in only half of sedated critically ill patients within two days [61]. The WEAN SAFE study showed in 5869 patients that deep sedation was independently associated with failure of liberation from the ventilator [62].

Of note, the association between deep sedation and delayed ventilator liberation was shown for the most widely used intravenous sedatives, propofol and midazolam, both of which typically suppress respiratory drive at deep sedation levels [5, 63]. Clinical trials have shown that sedatives with a low impact on respiratory drive, such as dexmedetomidine, may support liberation from the ventilator [64], presumably due to improved patient-ventilator synchrony and better preservation of diaphragm activity with dexmedetomidine than with propofol [63, 65, 66]. Although other factors, such as wake-up times and neurocognitive recovery after the discontinuation of sedation surely contribute, evidence accumulates that sedation-induced impairment of spontaneous breathing efforts delays ventilator liberation. Better preservation of respiratory drive with volatile anesthetics than with propofol or midazolam sedation, as outlined in detail in the previous chapter, suggests that patients with marginal or no effort could benefit from sedation with volatile anesthetics [52]. However, those with excessive effort may benefit from the strong respiratory depressant effects of propofol [5, 63].

In addition to better control of respiratory drive and effort, volatile anesthetics are eliminated through exhalation, independent of frequently impaired kidney and liver function in critically ill patients. The possibility of monitoring exhaled concentrations further allows for tight control of sedation depth and helps to predict awakening. Clinical trials confirmed that this translates into short wake-up times and early cognitive recovery upon cessation, even after deep or prolonged periods of inhaled sedation [40, 67]. The Sedaconda trial revealed that the median wake-up was significantly faster after isoflurane than propofol sedation on day 2 (20 min [IQR 10–30] vs 30 min [11–120]; *p* = 0.001). Subgroup and post hoc analyses revealed that isoflurane sedation increases the number of ICU- and delirium-free days, although a benefit for ventilator liberation remains unclear [68, 69].

Taken together, inhaled sedation with volatile anesthetics in combination with short-acting opioids may allow for more precise control of the respiratory pattern (i.e., effort and rate) and faster cognitive recovery upon discontinuation, with intriguing benefits for lung and diaphragm protection and early ventilator liberation.

### Technical limitations of inhaled sedation

**Fig. 5:**
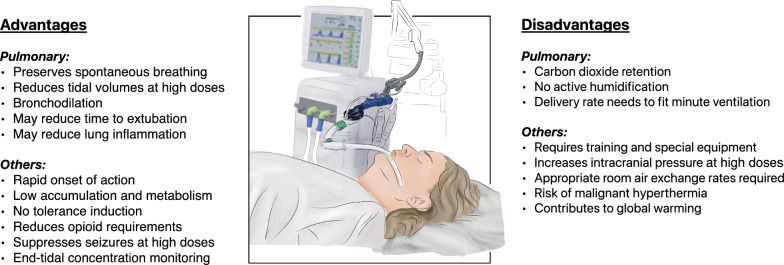
Advantages and disadvantages of inhaled sedation in the ICU. *Graphic design by Marco Rosetti*

In the following, we focus on ventilation-related limitations of inhaled sedation. A comprehensive list of advantages and disadvantages, which were discussed in various recent review articles [70–74], are presented in Fig. 5.

Reflection systems allow the efficient administration of volatile anesthetics via open-circuit ventilators. The technical details of three available commercial systems have been described elsewhere (Sedaconda ACD-S and ACD-L by Sedana Medical, Danderyd, Sweden; and Mirus®, Medcaptain, Nijmegen, The Netherlands [75]). In short, volatile anesthetic is either directly injected into the inspired breathing gas or infused into a hollow rod called the evaporator [76, 77]. An anesthetic reflector, inserted between the Y-piece and the patient, adsorbs the expired anesthetic and releases it during subsequent inspiration in a process called reflection [78]. Approximately 90% of the volatile anesthetic is reflected under dry conditions [77], which is reduced to roughly 80% under the influences of humidity and carbon dioxide, meaning that approximately 20% is lost during exhalation [79]. These losses are directly proportional to minute ventilation, and large changes in minute ventilation may require adjustments in the anesthetic infusion rate to keep the end-tidal concentration stable. As a rule of thumb, an isoflurane infusion rate of 3 mL/h with a minute ventilation of 6 L/min will yield a concentration of 0.5 Vol% in steady state (3 divided by 6 equals 0.5). One commercial device (Mirus) automatically adjusts the anesthetic infusion rate to maintain the end-tidal concentration at a set target value [76], although the end-tidal concentration does not correlate well with the clinically assessed sedation depth.

**Fig. 6:**
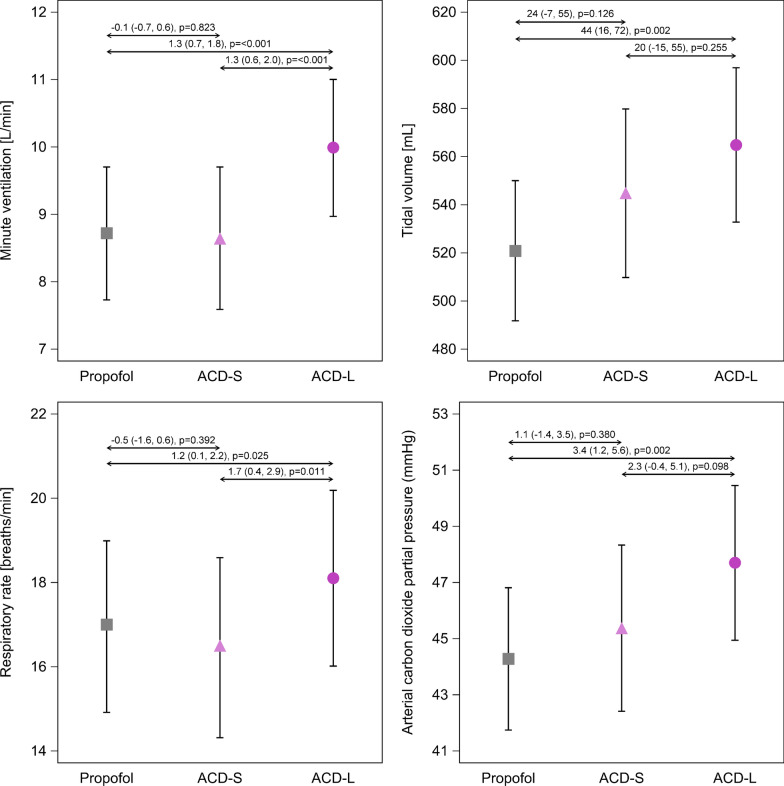
Comparison of ventilation parameters between propofol sedation (under ventilation with heat and moisture exchangers) and isoflurane sedation utilizing anesthetic conserving devices with different internal volumes from an a priori defined substudy of the Sedaconda trial. Propofol: n = 151; isoflurane, ACD-S: n = 64, ACD-L: n = 86. Data are presented as means and 95% confidence intervals (95%CI). Effect sizes are presented as average differences (95%CI) calculated by linear mixed effects models. ACD-S, anesthetic conserving device with 50 mL internal volume. ACD-L, anesthetic conserving device with 100 mL internal volume. *Reprinted under the terms of the Creative Commons Attribution License from* [53]

All reflectors increase dead space ventilation, first because of their internal volume (50 mL for ACD-S, 100 mL for ACD-L and Mirus) and second because of partial carbon dioxide reflection [80]. The additional increase in tidal volume needed to overcome this effect has been called reflective dead space and can reach 35–40 mL with a Sedaconda ACD-L [80–82] or 25 mL with Sedaconda ACD-S and Mirus devices [80, 81]. In laboratory studies, when using dry conditions without volatile anesthetics, the extent of carbon dioxide reflection may be highly overestimated, which has led some researchers to caution against the use of these devices in ARDS patients [54, 83]. However, in a substudy of the SEDACONDA trial, the use of the larger Sedaconda ACD-L was only associated with slightly greater arterial carbon dioxide partial pressures (3.4 mmHg), respiratory rates (1.2 bpm) and tidal volumes (44 mL) than ventilation with heat and moisture exchangers with internal volumes of 35 mL, whereas no difference was detected between ventilation with the smaller Sedaconda ACD-S and heat and moisture exchangers [53] (Fig. 6).

According to their technical specifications, Sedaconda ACD-S and Mirus are limited to use with tidal volumes of at least 200 mL, and Sedaconda ACD-L with tidal volumes of at least 300 mL. All reflection systems comprise passive humidification with low moisture loss (approximately 5 mg of water per liter of breathing gas) [84]. They cannot be combined with active humidification. Nebulizers can be connected between the reflector and the patient; however, some medications may bind to the reflector and increase resistance, in which case the reflector needs to be exchanged.

The use of anesthetic reflectors in patients undergoing extracorporeal membrane oxygenation is possible under consideration of the technique’s inherent limitations [85, 86]. With drastically reduced minute ventilation, volatile anesthetic administration rates must be similarly reduced to avoid overdosing [87]. If the tidal volume is less than 100 mL, gas monitoring of the end-tidal concentration will be inaccurate, and the sedation depth can only be monitored clinically. Modern membrane oxygenators made of polymethyl-pentene are not permeable to volatile anesthetics. Thus, volatile anesthetics can currently only be administered and eliminated via the lung.

## Conclusions

Safe spontaneous breathing efforts are crucial for preventing diaphragm disuse atrophy in invasively ventilated critically ill patients. Clearly, the approach to sedation in the ICU should move from a “one-sedative-fits-all” model towards an individualized strategy that considers the patient’s respiratory drive and effort as a means for achieving lung- and diaphragm-protective sedation and ventilation.

Inhaled sedation with volatile anesthetics compared to common intravenous alternatives offers superior preservation of respiratory drive with the potential to prevent diaphragm disuse atrophy. Concurrently, higher doses of volatile anesthetics reduce the size of spontaneously generated tidal volumes, presenting an opportunity to mitigate lung stress and strain. Inhaled sedation may thus allow for titrating respiratory drive to facilitate lung- and diaphragm-protective sedation and help to expedite liberation from the ventilator.

Further research is needed to understand the precise role of inhaled sedation with volatile anesthetics for modulation of respiratory drive and effort and how these effects translate into clinical outcomes.

## Data Availability

No datasets were generated or analysed during the current study.
